# Surgical strategies for hepatocellular carcinoma located in the left lateral lobe: A propensity score‐matched and prognostic nomogram study

**DOI:** 10.1002/cam4.3894

**Published:** 2021-05-01

**Authors:** Jingwen Zou, Shaohua Li, Qiaoxuan Wang, Jie Mei, Lianghe Lu, Wenping Lin, Yuhua Wen, Yuechao Li, Wei Wei, Rongping Guo

**Affiliations:** ^1^ Department of Liver Surgery Sun Yat‐sen University Cancer Center Guangzhou P. R. China; ^2^ State Key Laboratory of Oncology in South China Collaborative Innovation Center for Cancer Medicine Guangzhou P. R. China; ^3^ Department of Radiation Oncology Sun Yat‐sen University Cancer Center Guangzhou P. R. China

**Keywords:** cancer management, hepatocellular carcinoma, nomogram, surgery, survival

## Abstract

**Purpose:**

For hepatocellular carcinoma (HCC) located in the left lateral lobe, the optimal surgical procedure is still controversial. This study aimed to optimize surgical strategies and to construct a nomogram to predict the postoperative survival of patients with HCC.

**Methods:**

Between 1 January 2005 and 30 September 2018, a total of 493 patients were enrolled. Propensity score matching (PSM) was performed between the left lateral lobectomy (LLL) and left hepatectomy (LH) groups (1:1). The study endpoints were overall survival (OS), recurrence‐free survival (RFS), and safety. A nomogram was generated using a multivariate Cox proportional hazards model. The discriminative ability and calibration of the nomogram were evaluated using C‐statistics and calibration plots.

**Results:**

After matching, 87 pairs were included. The LH group had better 1‐, 3‐, and 5‐year OS rates than the LLL group (88%, 73%, and 69% vs. 73%, 57%, and 49%, respectively; *p* = 0.017). The 1‐, 3‐, and 5‐year RFS rates of the LH group were similar to those of the LLL group (64%, 49%, and 46% vs. 63%, 51%, and 42%, respectively; *p* = 0.652). There were no significant differences in postoperative complications. Eight factors were integrated into the nomogram and it had good discriminative ability and calibration.

**Conclusion:**

Our data revealed that compared to LLL, LH may result in better OS and have similar postoperative complications for HCC. The nomogram may serve as a practical tool for the individual prognostic evaluation of patients with HCC.

## Graphical abstract

Left hepatectomy is a more feasible and safer surgical approach for the treatment of HCC in the left lateral lobe. The proposed nomograms can provide patient‐specific survival information for patients with HCC in the left lateral lobe after surgery.

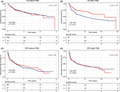

## 1 INTRODUCTION

Worldwide, hepatocellular carcinoma (HCC) was estimated to be the sixth most commonly diagnosed cancer and the fourth leading cause of cancer death in 2018.^1^ Surgical resection and liver transplantation are the first‐line curative‐intent therapies for the early and intermediate stages of HCC, respectively.^2^, ^3^, ^4^ Unfortunately, even with radical surgical resection, the 5‐year recurrence rates after surgery are still as high as 70% to 80%, severely limiting the long‐term survival of HCC patients.^5^, ^6^ Achieving long‐term survival for the early and intermediate stages of HCC remains a big challenge. Thus, it is critical to optimize the present treatment strategies to further improve the long‐term survival of patients with resectable HCC.

In recent years, studies on HCC located in the left lateral lobe have largely focused on surgical techniques. The laparoscopic approach has become similar to open surgery in many ways. One of the major advancements in laparoscopic liver resection is anatomic liver resection, including major and minor resection. Laparoscopic left lateral lobectomy (LLL) has been associated with shorter hospital stay and reduced overall morbidity compared to open LLL.^7^, ^8^, ^9^ Although the feasibility and safety of laparoscopic LLL and laparoscopic left hepatectomy (LH) have been widely confirmed, whether the range of excision extension for HCC located in the left lateral lobe can reduce the postoperative recurrence rate and improve the long‐term survival requires further verification. It may be time to consider changing the standard procedures for the treatment of HCC in the left lateral lobe in selected patients.

At present, for HCC located in the left lateral lobe, LLL or LH is generally performed. The average volume ratios of the left lateral segment, left medial segment, caudate lobe, right anterior segment, and right posterior segment were 17%, 14%, 2%, 37%, and 30%, respectively.^10^ The volume of the left liver is relatively small. However, because of the very frequent underlying liver disease, namely fibrosis and above all cirrhosis, resection has two contradictory aims: to be curative, with a safe tumor‐free margin, and to preserve as much functioning liver parenchyma as possible. Therefore, we question whether LH for HCC located in the left lateral lobe will bring better survival benefits to patients. On the one hand, LH with the extent of surgical resection ranging from Couinaud's segment II to IV will lead to more obvious liver function impairment in patients after the operation than LLL with the resection extent ranging from Couinaud's segment II to III. On the other hand, patients who undergo LH may achieve better long‐term survival because of the thoroughness of the operation. Thus, it is particularly important to identify patients who may benefit from LH. To the best of our knowledge, there have been no studies comparing the outcomes of LLL and LH. Propensity score matching (PSM) is a method proposed to overcome selection bias and increase the level of evidence in nonrandomized observational studies.

Therefore, this study was designed with the aim of further optimizing surgical decision‐making and improving patient prognosis. We also attempted to create and internally validate a nomogram to predict postoperative survival.

## 2 METHODS

### 2.1 Patients

From 1 January 2005 to 30 September 2018, all patients who underwent hepatic resection at the authors’ institution were consecutively included and retrospectively analyzed. The inclusion criteria were as follows: (1) HCC confirmed pathologically; (2) patients with HCC located in the left lateral section who were initially treated with LLL or LH; and (3) patients with complete clinical and follow‐up data. The exclusion criteria were as follows: (1) distant metastasis prior to the operation; (2) macroscopically positive [R2] or microscopically positive [R1] resection margin; (3) patients who had undergone any antitumor treatment modality before surgery; (4) patients with multiple primary cancers; and (5) patients who died within 30 days of surgery. The last follow‐up date was 31 December 2019.

The institutional review board of our department approved this study. All patients had signed informed consent prior to surgery.

### 2.2 Treatment and follow‐up

The hepatic resection procedure has been described in detail in a previous study.^11^ All patients included in our study underwent anatomic hepatectomy. LLL was defined as the systematic removal of II to III Couinaud's segment. LH was defined as the systematic removal of II to IV Couinaud's segment. Intraoperative ultrasonography was routinely performed to evaluate the number, size, and location of the lesions. The small portal branches supplying the liver parenchyma up to the aimed transection plane were punctured with ultrasound guidance and injected with dye, and then liver subsegmentectomy was performed along the dividing line defined by the injection dye. Pringle's maneuver was routinely used with a clamp/unclamp time of 10 min/5 min. The first follow‐up was carried out 1 month after the operation, then every 2–3 months within the first 2 years, and every 6–12 months afterward. For the follow‐up, measurements of serum alpha‐fetoprotein (AFP) level, hepatitis B virus (HBV) DNA load, liver and kidney function tests, and imaging examinations (abdominal contrast‐enhanced computed tomography (CT) or magnetic resonance imaging (MRI) and chest CT depending on the disease) were performed.^12^ When there was tumor recurrence during follow‐up, reoperation, local ablation, transarterial chemoembolization (TACE), radiotherapy, and chemotherapy were given according to the clinical practice guidelines^3^ and the wishes of the patients.

### 2.3 Study endpoints

The primary endpoint was overall survival (OS). The secondary endpoints included recurrence‐free survival (RFS), intraoperative outcomes (operative time, blood loss, and blood transfusion), and incidence of postoperative complications. Postoperative morbidity was defined as events that occurred within the first 60 days after surgery and was graded using the Clavien–Dindo classification.^13^ OS was defined as the time from the date of surgery to either the date of death or last follow‐up, while RFS was defined as the time from the date of surgery to the date of first recurrence, death, or last follow‐up.

### 2.4 Propensity score matching

PSM analysis was used to reduce the bias in treatment selection. The patients in the LLL and LH groups were matched using the propensity score method as described by Rubin and Rosenbaum.^14^, ^15^ The propensity score for an individual was calculated given the covariates of age, sex, tumor location, tumor number, tumor size, macrovascular invasion, Child‐Pugh classification, and preoperative serum AFP level using a logistic regression model. Thereafter, we applied 1:1 nearest neighbor matching with a caliper of 0.05 and without replacement to ensure that conditional bias was minimized.^16^

### 2.5 Prognostic nomogram

The enrolled patients were randomly grouped into the derivation set (*n* = 247) and the validation set (*n* = 246). Variables selected by multivariable Cox proportional hazards regression analyses as well as the demographic and tumor characteristics with clinical importance were incorporated into the nomogram to predict the probability of 1‐, 3‐, and 5‐year OS. The C‐statistic was used to assess the predictive accuracy for individual outcomes (discrimination ability) as proposed by Harrell et al.^17^ A calibration plot was used to evaluate the accuracy of the point estimates of the survival function (calibration).

### 2.6 Statistical analysis

Statistical analysis was performed using IBM SPSS Statistics version 25.0 (SPSS Inc.) and R software version 3.6.3 (The R Foundation for Statistical Computing) with the “survival”, “survminer”, “rms”, “ggsci”, and “forestplot” packages. Continuous variables are presented as mean ± standard deviation (SD) or median and interquartile range (IQR). Non‐normally distributed data were analyzed using the Mann–Whitney U test, and normally distributed data were compared using Student's *t* test. Categorical variables are presented as numbers (%) and were compared using the *χ* ^2^ test. Single ordinal contingency data were analyzed using the Kruskal–Wallis *H* test. Survival curves before and after PSM were depicted using the Kaplan–Meier method and compared using the log‐rank test. Multivariable Cox proportional hazards regression analyses were then performed to adjust for the other prognostic factors that were associated with OS and RFS. The candidate variables (*p* < 0.05) determined by univariate analysis were introduced into the multivariate Cox regression analysis. To investigate the effect of the surgical strategies (LLL or LH) on survival considering the potential confounders, the surgical strategies were included in the multivariate Cox regression analysis, regardless of whether their *p*‐value was statistically significant in univariate analysis. Statistical significance was set at *p* < 0.05.

## 3 RESULTS

**FIGURE 1:**
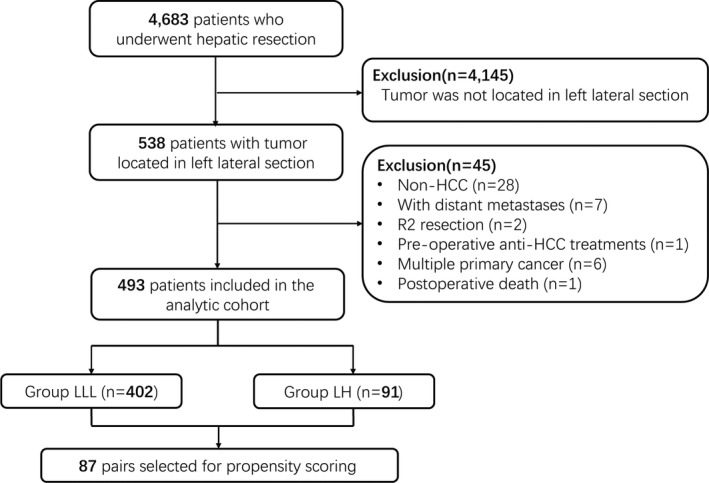
Flow chart of the study. HCC, hepatocellular carcinoma; LH, left hepatectomy; LLL, left lateral lobectomy; R2, patients with a macroscopically positive resection margin

During the study period, 4683 patients underwent hepatic resection. Based on the inclusion and exclusion criteria, 493 patients were included in the analytic cohort. Among them, 402 patients underwent LLL, and 91 patients underwent LH. PSM created 87 pairs of patients who underwent LLL or LH (Figure 1).

### 3.1 Perioperative characteristics

The perioperative characteristics are listed in Table 1. In the LH group, more patients had tumors in both Couinaud's segment II and segment III (*p* < 0.001), more patients had three or more tumors (*p* = 0.011), and more patients had macrovascular invasion (*p* < 0.001). The maximum tumor size was larger in the LH group (*p* < 0.001). There were more patients in the LH group with Child‐Pugh class B (*p* = 0.006) and Barcelona Clinic Liver Cancer (BCLC) stages B and C (*p* < 0.001). The preoperative platelet count was higher in the LH group (*p* < 0.001). There were no significant differences in the other baseline characteristics between the two groups. Patients in the LH group had longer operative times (*p* < 0.001) and hepatic portal control times (*p* < 0.001). More patients in the LLL group underwent laparoscopic liver resection (*p* < 0.001). More patients in the LH group had microvascular invasion (*p* = 0.023). Intraoperative blood loss was higher in the LH group (*p* < 0.001). There were no significant differences in the other operative and postoperative data between the two groups. After PSM analysis, 87 matched pairs were selected from each group, and there was no significant difference in baseline characteristics between the two groups. Patients in the LH group had a longer operative time (*p* < 0.001), and more patients in the LH group underwent laparoscopic liver resection (*p* = 0.011) after PSM. There were no significant differences in the incidence or types of complications or Clavien–Dindo classification after surgery (Table 2). The postoperative hemato‐biochemical parameters after PSM are listed in Table S1. Compared to the LLL group, postoperative liver function damage was more obvious and AFP levels declined more rapidly in the LH group.

**TABLE 1:** Perioperative characteristics of the study patients before and after propensity score matching

	Before propensity matching			After propensity matching			
	Group LLL (*n* = 402)	Group LH (*n* = 91)	*p* value	Group LLL (*n* = 87)	Group LH (*n* = 87)	*p* value	
*Baseline characteristics*							
Age (year) (mean ± SD)	52 ± 12	50 ± 13	0.111	48.06 ± 11.53	49.16 ± 12.78		0.551
Gender (*n* (%))							
Male	330 (82.10%)	78 (85.70%)	0.408	73 (83.90%)	74 (85.10%)		0.834
Female	72 (17.90%)	13 (14.30%)		14 (16.10%)	13 (14.90%)		
HBsAg (*n* (%))							
Positive	359 (89.50%)	81 (89%)	0.885	79 (90.80%)	78 (89.70%)		0.798
Negative	42 (10.50%)	10 (11%)		8 (9.2%)	9 (10.30%)		
HBV DNA (IU/ml) (median (IQR))	2490 (0–225500)	700 (0–286000)	0.845	12100 (33.74–540500)	739 (0–277500)		0.179
Tumor location (*n* (%))							
S2	73 (18.20%)	6 (6.60%)	**<0.001**	8 (9.20%)	6 (6.90%)		0.396
S3	125 (31.10%)	14 (15.40%)		20 (23%)	14 (16.10%)		
S2&S3	204 (50.7%)	71 (78%)		59 (67.80%)	67 (77%)		
Tumor number (*n* (%))							
1	351 (87.30%)	71 (78%)	**0.011**	70 (80.50%)	69 (79.30%)		0.942
2	24 (6%)	5 (5.50%)		4 (4.60%)	5 (5.70%)		
≥3	27 (6.70%)	15 (16.50%)		13 (14.90%)	13 (14.90%)		
Tumor size (cm) (median (IQR))	5 (3–7)	7.5 (5–10)	**<0.001**	8 (4–12)	7 (5–10)		0.598
Macrovascular invasion (*n* (%))							
Present	21 (5.20%)	18 (19.80%)	**<0.001**	17 (19.50%)	15 (17.20%)		0.696
Absent	381 (94.80%)	73 (80.20%)		70 (80.50%)	72 (82.80%)		
Cirrhosis (*n* (%))							
None	159 (40.90%)	42 (46.70%)	0.492	37 (43.50%)	40 (46.50%)		0.360
Low grade	81 (20.80%)	14 (15.60%)		29 (34.10%)	13 (15.10%)		
Middle grade	122 (31.40%)	29 (32.20%)		18 (21.20%)	28 (32.6%)		
High grade	27 (6.90%)	5 (5.60%)		1 (1.20%)	5 (5.80%)		
ICGR15 (%) (median (IQR))	4 (2–6.50)	3.50 (1.80–6.05)	0.251	4 (2.25–6.85)	3.20 (1.70–6.20)		0.100
Child‐Pugh classification (*n* (%))							
A	397 (100%)	88 (96.70%)	**0.006**	87 (100%)	87 (100%)		NA
B	0	3 (3.30%)		0	0		
BCLC stage (*n* (%))							
A	335 (84.40%)	57 (62.60%)	**<0.001**	60 (69%)	57 (65.50%)		0.548
B	38 (9.60%)	16 (17.60%)		10 (11.5%)	15 (17.20%)		
C	24 (6%)	18 (19.80%)		17 (19.50%)	15 (17.20%)		
PRO AFP (ng/ml) (*n* (%))							
≥400	142 (36.30%)	34 (37.80%)	0.795	44 (50.60%)	32 (36.80%)		0.067
<400	249 (63.70%)	56 (62.20%)		43 (49.40%)	55 (63.20%)		
PRO WBC (×10^9^/L) (mean ± SD)	6.33 ± 2.12	6.71 ± 2.05	0.120	6.75 ± 2.56	6.65 ± 1.97		0.772
PRO Neutrophil (×10^9^/L) (mean ± SD)	3.83 ± 1.79	3.96 ± 1.59	0.510	4.18 ± 2.35	3.88 ± 1.49		0.318
PRO Hemoglobin (g/L) (mean ± SD)	143.18 ± 22.39	143.24 ± 16.17	0.984	139.76 ± 16.47	143.80 ± 16.01		0.102
PRO Platelet (×10^9^/L) (mean ± SD)	186.37 ± 76.34	222.88 ± 89.78	**<0.001**	202.91 ± 92.61	219.80 ± 89.85		0.224
PRO Total Bilirubin (µmol/L) (*n* (%))							
>20.5	58 (14.50%)	13 (14.30%)	0.965	12 (13.80%)	10 (11.50%)		0.648
≤20.5	343 (85.50%)	78 (85.70%)		75 (86.20%)	77 (88.50%)		
PRO ALT (U/L) (*n* (%))							
>40	133 (33.20%)	39 (42.90%)	0.080	34 (39.50%)	35 (40.70%)		0.876
≤40	268 (66.80%)	52 (57.10%)		52 (60.50%)	51 (59.30%)		
PRO Albumin (g/L) (mean ± SD)	43.18 ± 4.85	42.56 ± 4.19	0.262	43.08 ± 6.17	42.86 ± 3.96		0.780
PRO Creatinine (μmoI/L) (mean ± SD)	75.79 ± 17.73	73.17 ± 17.79	0.204	75.51 ± 18.77	73.46 ± 18.06		0.463
*Intraoperative and Postoperative Data*							
Operative time (min)*(median (IQR))	135 (110–165)	180 (150–210)	**<0.001**	150 (120–180)	180 (150–210)		**<0.001**
Hepatic portal control (min)*(median (IQR))	0 (0–5.50)	4.50 (0–16)	**<0.001**	4.25 (0–15)	4.50 (0–16.25)		0.528
Laparoscopic approach (*n* (%))	111 (27.6%)	8 (8.80%)	**<0.001**	0	8 (9.20%)		**0.011**
Blood loss (ml)* (median (IQR))	100 (100–200)	200 (200–400)	**<0.001**	200 (100–400)	200 (200–400)		0.346
Blood transfusion (*n* (%))	28 (7.10%)	8 (8.80%)	0.567	12 (14.30%)	7 (8%)		0.194
Margin width (cm)*(median (IQR))	2 (1–3)	2 (1.15–3)	0.353	2 (1–2)	2 (1–3)		0.123
Microvascular invasion (*n* (%))	125 (44%)	43 (58.90%)	**0.023**	17 (70.80%)	41 (57.70%)		0.256
Histology grade (*n* (%))							
I	23 (5.80%)	3 (3.30%)	0.442	7 (8.10%)	3 (3.40%)		0.586
II	204 (51.40%)	46 (50.50%)		41 (47.70%)	44 (50.60%)		
III	170 (42.80%)	42 (46.20%)		38 (44.20%)	40 (46%)		

Abbreviations: AFP, alpha‐fetoprotein; ALT, alanine aminotransferase; HBV, hepatitis B virus; ICGR15, indocyanine green retention rate at 15 min; LH, left hepatectomy; LLL, left lateral lobectomy; PRO, Preoperative; WBC, white blood cell.

Bold values indicate a statistically significant difference with a *p* value < 0.05.

**TABLE 2:** Postoperative complications of the two groups after propensity score matching

	Group LLL (*n* = 87)	Group LH (*n* = 87)	*p* value
Complications	9 (10.34%)	13 (14.90%)	0.362
Complication type			
Fever	6 (6.90%)	5 (5.70%)	0.141
Nausea and vomiting	1 (1.10%)	3 (3.40%)	
Anhelation	0	3 (3.40%)	
Intestinal obstruction	1 (1.10%)	0	
Atrial fibrillation	1 (1.10%)	0	
Abdominal infection	0	1 (1.10%)	
Urinary tract infection	0	1 (1.10%)	
Clavien–Dindo grade			
I	7 (8%)	12 (13.80%)	0.411
II	2 (2.30%)	1 (1.10%)	
III‐V	0	0	

Abbreviations: LH: left hepatectomy; LLL: left lateral lobectomy.

### 3.2 Survival analysis

**FIGURE 2:**
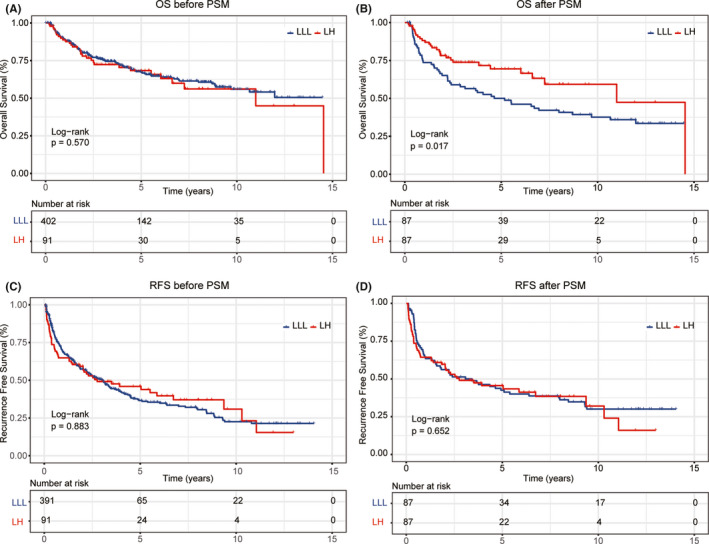
Kaplan–Meier curves of survival rates for HCC patients who underwent LLL or LH. (A) Overall survival rates for all study patients before propensity score matching analysis; (B) Overall survival rates for patients after propensity score matching analysis; (C) Recurrence‐free survival rates for all study patients before propensity score matching analysis; (D) Recurrence‐free survival rates for patients after propensity score matching analysis. LH: left hepatectomy; LLL, left lateral lobectomy

During a median follow‐up period of 71.5 months, 129 (32.09%) patients in the LLL group and 31 (34.07%) patients in the LH group died. There were 214 patients in the LLL group (54.73%) and 54 patients in the LH group (59.34%) with tumor recurrence. The 1‐, 3‐, and 5‐year OS rates in the LH group (88%, 72%, and 68%, respectively) were similar to those in the LLL group (88%, 75%, and 67%, respectively) (*p* = 0.570) (Figure 2A). There was no significant difference in RFS rates between the two groups: the 1‐year, 3‐year, and 5‐year RFS rates in the LH group were 65%, 49%, and 46%, respectively, and the RFS rates in the LLL group were 68%, 50%, and 36%, respectively (*p* = 0.883) (Figure 2C).

**FIGURE 3:**
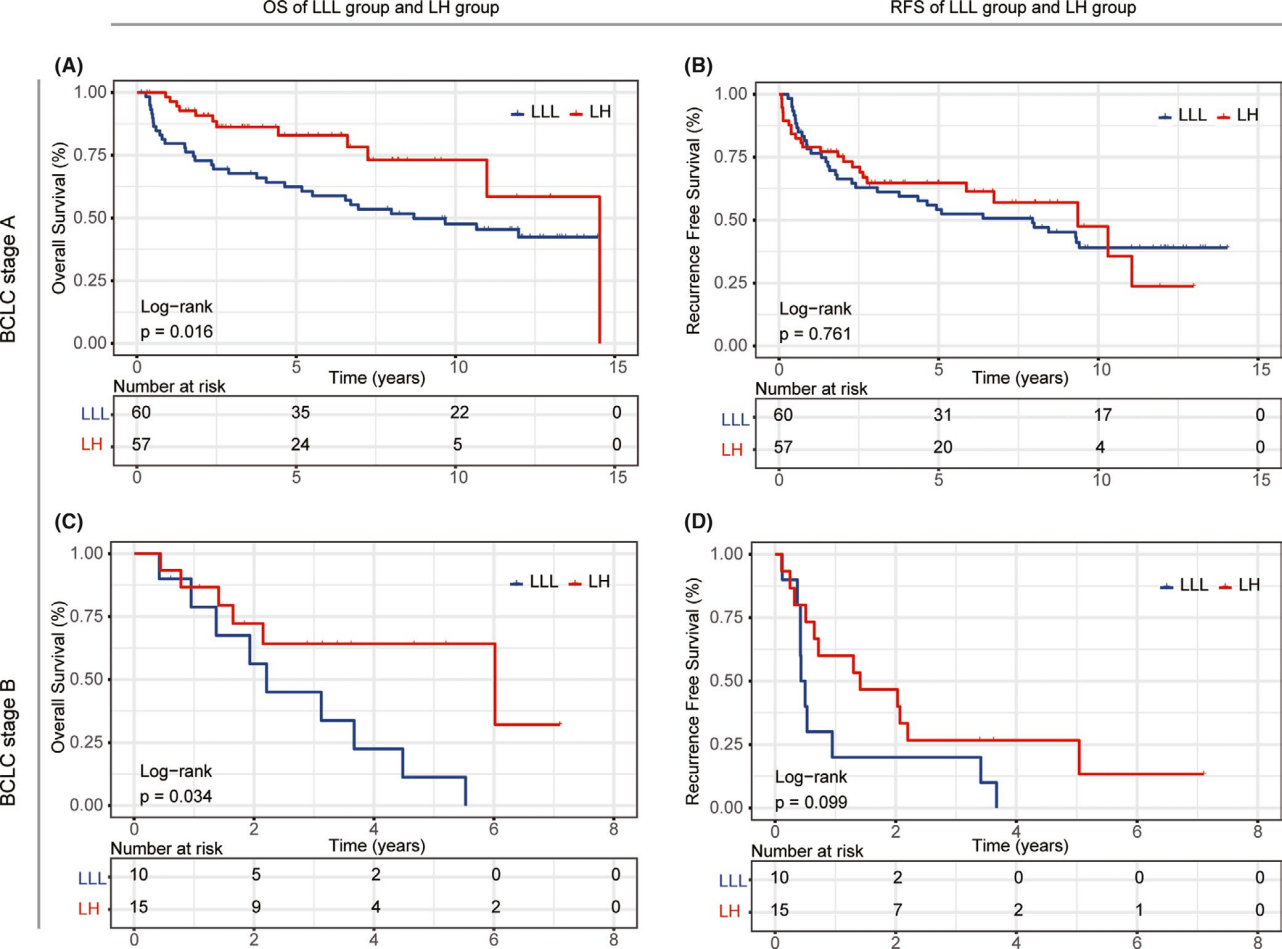
Kaplan–Meier curves of stage‐specific HCC patients who underwent LLL or LH. (A) BCLC stage‐A‐specific overall survival; (B) BCLC stage‐B‐specific overall survival; (C) BCLC stage‐A‐specific recurrence‐free survival; (D) BCLC stage‐B‐specific recurrence‐free survival. LH: left hepatectomy; LLL, left lateral lobectomy

**FIGURE 4:**
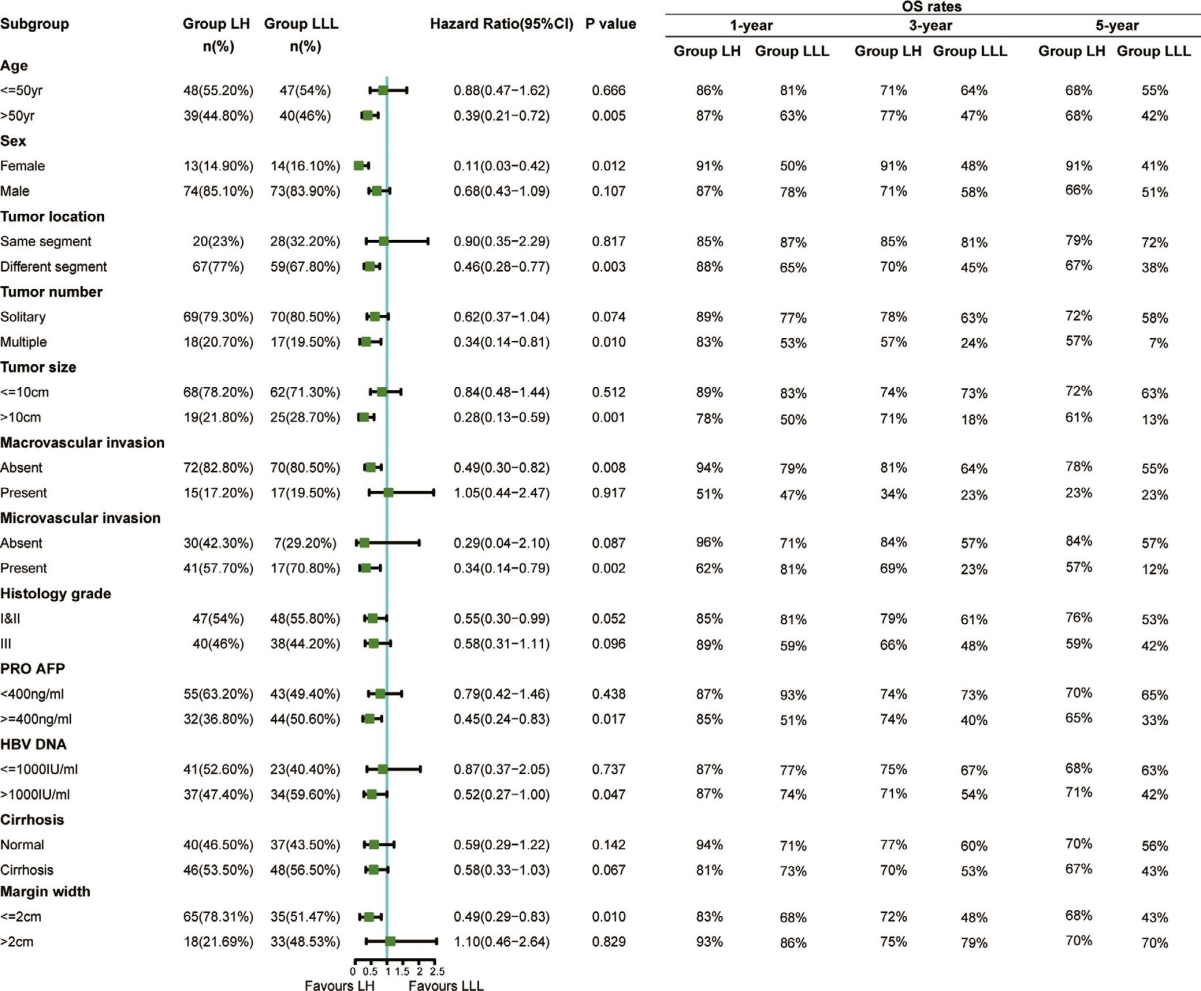
Forest plot for overall survival of patients after propensity score matching analysis. LH: left hepatectomy; LLL, left lateral lobectomy POD: preoperative

After PSM analysis, patients in the LH group showed better OS than those in the LLL group. The 1‐, 3‐, and 5‐year OS rates in the LH group were 88%, 73%, and 69%, respectively, and those in the LLL group were 73%, 57%, and 49%, respectively (*p* = 0.017) (Figure 2B). However, there was no statistically significant difference in RFS rates between the two groups (1‐year, 3‐year, and 5‐year RFS rates were 63%, 49%, and 46%, respectively, in the LH group, and 63%, 51%, and 42%, respectively, in the LLL group; *p* = 0.652) (Figure 2D). Eighteen (20.70%) patients in the LLL group and 31 (35.60%) patients in the LH group did not have disease recurrence during the follow‐up period (*p* = 0.036). Further analysis of recurrence pattern showed that there was no significant difference between the two groups (Table 3). When patients with BCLC stage‐A, patients in the LH group had better OS (*p* = 0.016), while RFS was comparable between the two groups (*p* = 0.761). When patients with BCLC stage‐B, patients in the LH group had better OS (*p* = 0.034) and RFS became obvious between the two groups (*p* = 0.099) (Figure 3). Subgroup analysis of OS showed that specific subgroups may benefit most from LH, such as older than 50 years, female sex, tumor located in the adjacent two segments, multiple tumors, maximum tumor size larger than 10 cm, presence of microvascular invasion, preoperative AFP ≥400 ng/ml, HBV DNA >1000 IU/ml, and margin width ≤2 cm (Figure 4). To explore the potential effect on survival of the surgical methods, namely laparoscopic lobectomy or open lobectomy, we excluded the patients who underwent laparoscopic liver resection and re‐do the PSM analysis. The results show that the two groups had different OS and similar RFS (Figure S1).

**TABLE 3:** Postoperative recurrence rate and pattern of patients in the PSM cohort

	Group LLL	Group LH	*p* value
Follow‐up data			
Recurrence‐free	18 (20.70%)	31 (35.60%)	**0.036**
Recurrence	57 (65.50%)	51 (58.60%)	
Lost to follow‐up	12 (13.80%)	5 (5.70%)	
Recurrence pattern			
Intrahepatic recurrence	14 (60.90%)	29 (64.4%)	0.772
Extrahepatic metastasis	5 (21.70%)	9 (20%)	
Lung	4	6	0.867
Lymph node (s)	0	1	
Lung + bone	1	0	
Lung + lymph node (s)	0	1	
Lung + adrenal gland (s)	0	1	
Intrahepatic recurrence & Extrahepatic metastasis	4 (17.40%)	7 (15.60%)	0.846

Abbreviations: LH, left hepatectomy; LLL, left lateral lobectomy; PSM, propensity score matching.

Bold values indicate a statistically significant difference with a *p* value < 0.05.

### 3.3 Risk factor analysis

In the PSM cohort, LLL, multiple tumors, tumor size >10 cm, presence of macrovascular invasion, and PRO AFP ≥400 ng/ml were considered significant risk factors (*p* < 0.05) for OS in univariate analysis (Table 4). Age >50, tumors located in different segments, multiple tumors, tumor size >10 cm, presence of macrovascular invasion, histological grade III, and PRO AFP ≥400 ng/ml were considered significant risk factors (*p* < 0.05) for RFS in univariate analysis (Table 5). Multivariate analysis showed that LLL, multiple tumors, tumor size >10 cm, and presence of macrovascular invasion were independent risk factors for OS after PSM (*p* < 0.05), while age >50, multiple tumors, and the presence of macrovascular invasion were independent risk factors for RFS after PSM (*p* < 0.05).

**TABLE 4:** Univariate and multivariate analyses of the relative risk of overall survival after propensity score matching

Variable	Univariate		Multivariate	
	HR (95% CI)	*p* value	HR (95% CI)	*p* value
Group (LLL vs. LH)	0.567 (0.354–0.908)	**0.018**	0.562 (0.349–0.903)	**0.017**
Age (year) (≤50 vs. >50)	1.380 (0.884–2.153)	0.156		
Sex (female vs. male)	1.328 (0.663–2.661)	0.423		
Tumor location (Same segment vs. different segment)	1.665 (0.997–2.780)	0.051		
Tumor number (solitary vs. multiple)	2.701 (1.615–4.517)	**<0.001**	2.421 (1.417–4.137)	**0.001**
Tumor size (cm) (≤10 vs. >10)	2.406 (1.503–3.852)	**<0.001**	2.029 (1.236–3.331)	**0.005**
Macrovascular invasion (absent vs. present)	3.485 (2.087–5.818)	**<0.001**	2.672 (1.527–4.677)	**0.001**
Histology grade (I&II vs. III)	1.335 (0.857–2.081)	0.202		
PRO AFP (ng/ml) (<400 vs. ≥400)	1.875 (1.203–2.922)	**0.005**	1.295 (0.800–2.096)	0.293
HBV DNA (IU/ml) (≤1000 vs. >1000)	1.658 (0.967–2.844)	0.066		
Cirrhosis (normal vs. cirrhosis)	1.398 (0.881–2.219)	0.155		
Margin width (cm) (≤2 vs. >2)	0.686 (0.411–1.143)	0.148		

Note

The former in parentheses is the reference.

Abbreviations: AFP, alpha‐fetoprotein; HBV, hepatitis B virus; ICGR15, indocyanine green retention rate at 15 min; LH, left hepatectomy; LLL, left lateral lobectomy; PRO, Preoperative.

Bold values indicate a statistically significant difference with a *p* value < 0.05.

**TABLE 5:** Univariate and multivariate analyses of the relative risk of recurrence‐free survival after propensity score matching

Variable	Univariate		Multivariate	
	HR (95% CI)	*p* value	HR (95% CI)	*p* value
Group (LLL vs. LH)	1.091 (0.744–1.600)	0.655	1.234 (0.823–1.849)	0.309
Age (yr) (≤50 vs. >50)	1.648 (1.126–2.411)	**0.010**	1.522 (1.034–2.239)	**0.033**
Sex (female vs. male)	1.371 (0.767–2.449)	0.287		
Tumor location (Same segment vs. different segment)	2.100 (1.320–3.340)	**0.002**	1.297 (0.766–2.196)	0.333
Tumor number (solitary vs. multiple)	3.166 (2.036–4.925)	**<0.001**	2.344 (1.465–3.751)	**<0.001**
Tumor size (cm) (≤10 vs. >10)	2.046 (1.359–3.081)	**0.001**	1.399 (0.886–2.209)	0.150
Macrovascular invasion (absent vs. present)	3.466 (2.213–5.430)	**<0.001**	2.756 (1.676–4.532)	**<0.001**
Histology grade (I&II vs. III)	1.582 (1.081–2.316)	**0.018**	1.158 (0.777–1.726)	0.472
PRO AFP (ng/ml) (<400 vs. ≥400)	1.775 (1.214–2.595)	**0.003**	1.278 (0.844–1.936)	0.246
HBV DNA (IU/ml) (≤1000 vs. >1000)	1.387 (0.890–2.162)	0.149		
Cirrhosis (normal vs. cirrhosis)	1.224 (0.827–1.813)	0.313		
Margin width (cm) (≤2 vs. >2)	0.678 (0.435–1.058)	0.087		

Note

The former in parentheses is the reference.

Abbreviations: AFP, alpha‐fetoprotein; HBV, hepatitis B virus; ICGR15, indocyanine green retention rate at 15 min; LH, left hepatectomy; LLL, left lateral lobectomy; PRO, Preoperative.

Bold values indicate a statistically significant difference with a *p* value < 0.05.

### 3.4 Prognostic nomogram

**FIGURE 5:**
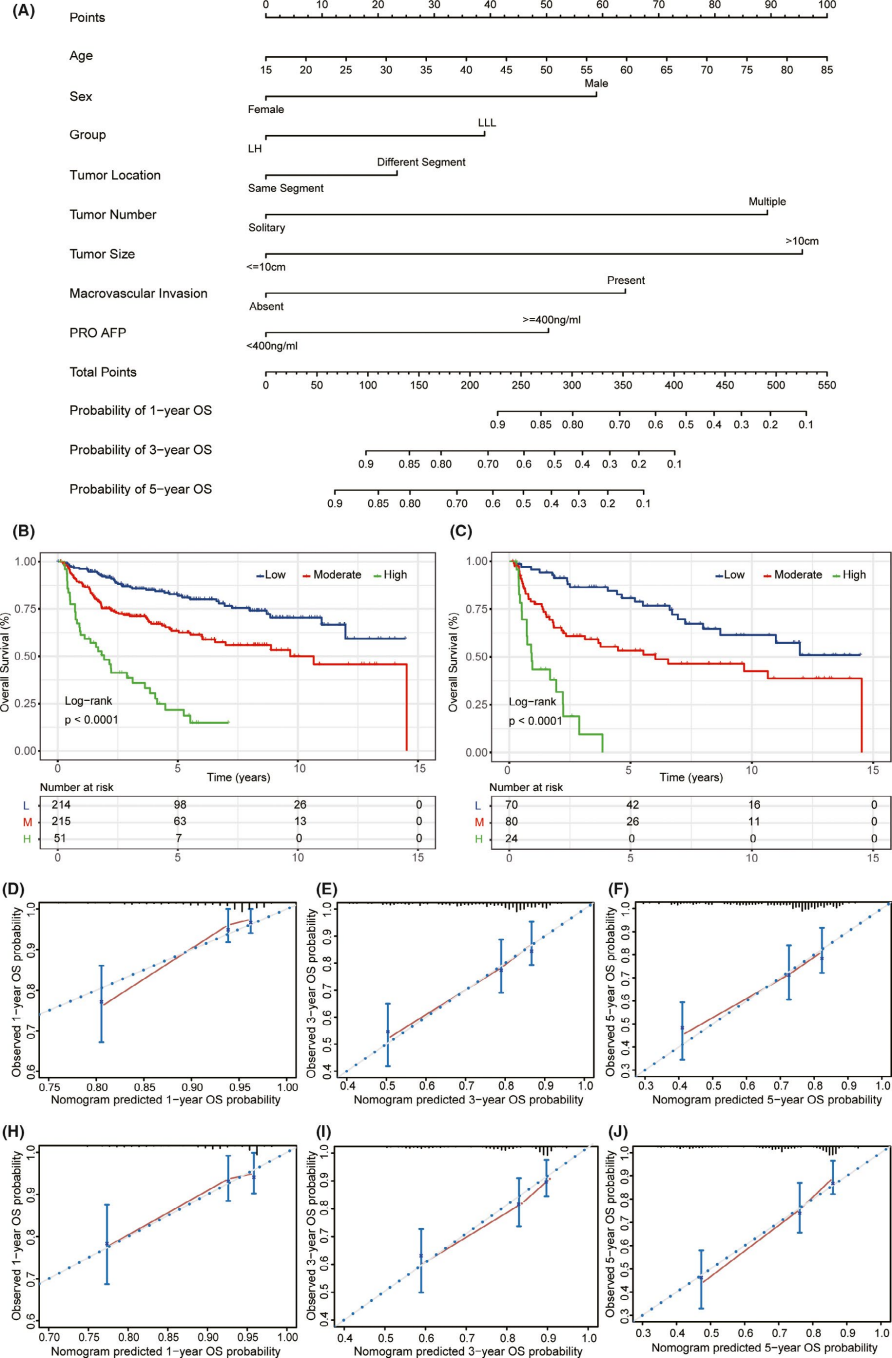
Nomogram (upper) and validation plot (middle and lower). (A) The nomogram to predict overall survival was developed based on eight prognostic factors; (B) Kaplan–Meier curves show survival of all study patients who underwent LH or LLL according to prognostic index of the model; (C) Kaplan–Meier curves for patients after propensity score matching analysis according to prognostic index of the model; (D–F) Calibration plots of derivation set; (H–J) calibration plots of validation set. LH: left hepatectomy; LLL, left lateral lobectomy; OS: overall survival; POD: preoperative

The nomogram for predicting OS was constructed based on the following eight prognostic factors: age, sex, group (LH or LLL), tumor location (same segment or different segments), tumor number (solitary or multiple), tumor size (≤10 or >10 cm), macrovascular invasion (absence or presence), and PRO AFP (<400 or ≥400 ng/ml) (Figure 5A). C‐statistic was used to assess the discriminative ability of the nomogram for OS. In the derivation set, the C‐statistic was 0.732, and in the validation set, it was 0.722. X‐tile software was used to further evaluate the discriminative ability of the model according to the prognostic index (PI). PI = 0.0146*Age (year) + 0.6005*Gender + (−0.3973)*Group +0.2382*Tumor Location +0.9111*Tumor Number + 0.9744*Tumor Size + 0.6531*Macrovascular Invasion + 0.5133*PRO AFP. Patients with a high PI had a substantially worse OS than those with low and moderate PI (*p* < 0.001) (Figure 5B,C). The calibration plots to predict 1‐, 3‐, and 5‐year OS showed good agreement between the nomogram predictions and the actual observations in both the derivation and validation sets (Figure 5D–J).

## 4 DISCUSSION

The survival of patients with HCC is generally poor, with a 5‐year OS rate of less than 15%. Hepatectomy remains the primary radical treatment for HCC patients, with a 5‐year survival rate of 40% to 70% after surgery.^18^, ^19^ It is difficult to compare the overall effect of LLL and LH in the treatment of HCC located in the left lateral lobe. It even could not conduct a randomized design in a prospective study. However, this is a controversial and an urgent clinical problem to be solved. Thus, we attempted to conduct the PSM analysis to perform a well‐matched and balanced comparison based on clinical factors that affected the results. In this retrospective study, the results showed that patients who underwent LH had a better OS than those who underwent LLL, and no increased risk of postoperative complications was identified. The better results in the LH group can be explained by two reasons. Firstly, the LH group had wider range of resection and was more likely to remove the potential micrometastases. Intrahepatic metastasis is a common site of HCC metastasis. It was reported that the presence of micrometastasis is associated with metastasis, recurrence, and unfavorable survival outcomes of the patients.^20^, ^21^ Secondly, for HCC located in the left lateral lobe, LH ensures a safe margin so that the primary lesions can be completely removed. However, it does not mean that all the patients with HCC located in the left lateral lobe should be offered LH. The results of subgroup analysis suggested that when patients with tumor located in the adjacent two segments, multiple tumors, maximum tumor size larger than 10 cm, preoperative AFP ≥400 ng/ml, HBV DNA >1000 IU/ml, and margin width ≤2 cm were more inclined to recommend LH.

Interestingly, contrary to our expectations, this study did not find a significant difference in RFS between the two groups. Although the difference in recurrence rate between the LLL and LH groups did not reach statistical significance, a markedly lower number of patients experienced recurrence in the LH group (LH 35.60% vs. LLL 20.70%). When patients with BCLC stage‐A HCC were compared, RFS was comparable between the two groups. In patients with BCLC stage‐B HCC, however, the survival benefit of the LH group became obvious. The 1‐ and 3‐year RFS were 60% and 27% in the LH versus 26% and 20% in the LLL group (*p* = 0.099). These data suggested that for BCLC stage‐A HCC, the choice of LLL or LH depends largely on the margin width and general condition of the patient. In addition, LH is more inclined to recommend for patients with BCLC stage‐B HCC. It was suggested that BCLC stage and margin width are two possible indicators for the selection of surgical strategies for HCC located in the left lateral lobe. Nevertheless, owing to relatively small sample size, there may still exist some residual confounding because of unmeasured or unknown confounders and biasing the results. Thus, our results need to be further confirmed by expanding the sample size and conducting multicenter research in the future, so as to obtain more definitive conclusions to guide clinical treatment.

In the PSM cohort, patients in the LH group had longer operative times than those patients in the LLL group. As the range of liver resection increases, the operation becomes more difficult and therefore results in a longer operative time.^10^ It is worth noting that all patients in the two groups had good liver function (Child‐Pugh grade A) after PSM. Therefore, HCC patients with sufficient liver reserve may obtain a survival benefit after undergoing LH without an increased incidence of postoperative complications.^22^, ^23^ However, it remains unclear whether the occurrence of postoperative complications will increase in patients with Child‐Pugh grade B after LH. Therefore, further research is needed to identify whether LH is equally safe and effective in other subgroups.

In previous studies, tumor number, tumor size, macrovascular invasion, and PRO AFP have been repeatedly confirmed to be associated with the prognosis of HCC.^24^, ^25^, ^26^ Thus, combining the results of multivariate analysis and subgroup analysis, group (LH or LLL), tumor number (solitary or multiple), tumor size (≤10 or >10 cm), and macrovascular invasion (absence or presence) were integrated into the nomogram to predict postoperative OS. In this study, we constructed and internally validated a prognostic nomogram with good discrimination and calibration. This nomogram is an accurate, repeatable, and individual prognostic tool for patients with HCC located in the left lateral lobe. All eight variables contained in the nomogram described above can be easily obtained in daily practice at no additional costs. This user‐friendly nomogram may allow physicians to easily calculate the survival risk at the individual level. In conclusion, this nomogram is convenient for clinical application and can help patients to determine whether to undergo LH or LLL by predicting the probability of OS, thus better guiding individualized treatment.

However, this study has a few limitations. First, its retrospective nature is the primary limitation. Although we applied PSM analysis to minimize the bias caused by retrospective studies, it was still inferior to prospective studies. Second, the HCC patients in this study were mostly infected with hepatitis B virus. Therefore, these data may not be suitable for HCC patients in Western countries, where HCC is more commonly caused by hepatitis C virus infection and alcohol consumption. Third, although the overall sample size of patients exceeded 300 (including 91 patients who underwent LH), the sample size was still relatively small, especially for the number of patients in the LH group. It was impossible to perform statistical analyses for some specific subgroups because of the small sample size. Finally, while a single‐center study undoubtedly allows for the standardization of the operative approach, the single‐center nature of this study is also a disadvantage because it cannot improve the generalizability of our research outcomes. To our knowledge, this is the first study to exclusively compare the outcomes of patients with HCC in the left lateral lobe who underwent curative‐intent LLL or LH. Some useful suggestions for the treatment of these patients have been put forward.

## 5 CONCLUSION

Compared to LLL, LH is a more feasible and safer surgical approach for the treatment of HCC in the left lateral lobe. The proposed nomograms can provide patient‐specific survival information for patients with HCC in the left lateral lobe after surgery.

## ETHICS APPROVAL

The institutional review board at our department approved this study (IRB approved number was B2019‐057–01).

## CONSENT TO PARTICIPATE

All patients signed an informed consent form before surgery.

## CONFLICT OF INTEREST

Conflict of interest relevant to this article was not reported.

## AUTHOR CONTRIBUTIONS

RG, JZ, SL, and WW designed this study. JZ, QW, LL, and JM analyzed and interpreted the patient data. JZ, WL, YW, and YL collected important background information and drafted the manuscript. All authors read and approved the final manuscript.

## Supporting information

Fig S1

Table S1

## Data Availability

The raw data of this study have been uploaded to the Research Data Deposit public platform (www.researchdata.org.cn) (No. RDDA2020001680).
