# Supplementary figures and images for: Surgical strategies for hepatocellular carcinoma located in the left lateral lobe: A propensity score‐matched and prognostic nomogram study
Source: Cancer Med. 2021 May 1;10(10):3274–87. doi: 10.1002/cam4.3894 (PMC8124126; doi:10.1002/cam4.3894)

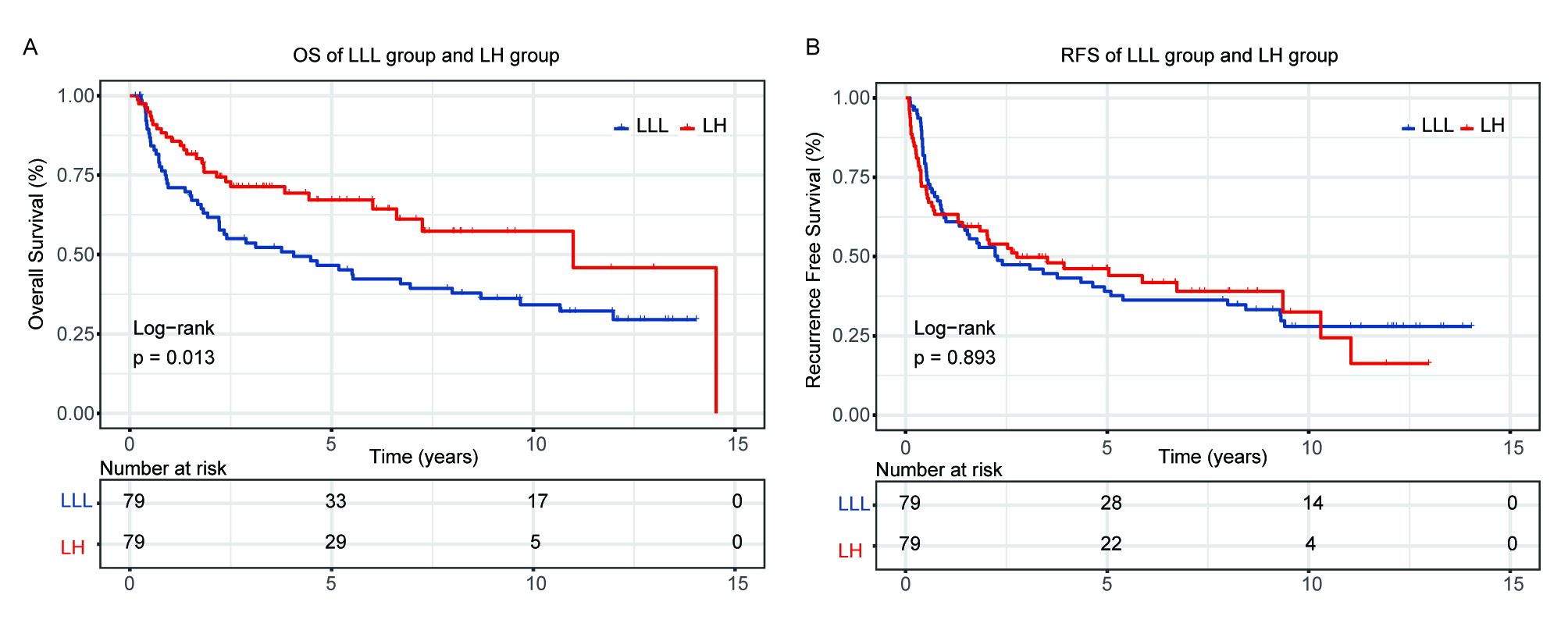

Supplement: Supplementary file 1 — Fig S1 [file CAM4-10-3274-s002.tif]
