# Supplementary material for: Surgical strategies for hepatocellular carcinoma located in the left lateral lobe: A propensity score‐matched and prognostic nomogram study
Source: Cancer Med. 2021 May 1;10(10):3274–87. doi: 10.1002/cam4.3894 (PMC8124126; doi:10.1002/cam4.3894)
Supplement: Supplementary file 2 — Table S1 [file CAM4-10-3274-s001.docx]

Table S1. Postoperative hemato-biochemical parameters after propensity score matching.

| Characteristics | Group LLL (n=87) | Group LH (n=87) | P value |
| --- | --- | --- | --- |
| POD1 WBC (× 10^9^/L) (mean, SD) | 12.82±4.09 | 13.92±4.11 | 0.155 |
| POD1 Neutrophil (× 10^9^/L) (mean, SD) | 10.88±4.08 | 12.02±3.40 | 0.125 |
| POD1 Hemoglobin (g/L) (mean, SD) | 125.10±17.57 | 131.65±24.22 | 0.115 |
| POD1 Platelet (× 10^9^/L) (mean, SD) | 159.58±93.83 | 166.04±70.39 | 0.663 |
| POD1 Total Bilirubin (µmol/L) (n, %) | 17.20(11.95-23.75) | 19.20(13-22.63) | 0.759 |
| POD1 ALT (U/L) (n, %) | 144.45(98.78-226.33) | 184.70(141.30-302.20) | **0.007** |
| POD1 Albumin (g/L) (mean, SD) | 34.49±5.17 | 39.03±36.92 | 0.430 |
| POD1 Creatinine (μmoI/L) (mean, SD) | 69.71±15.82 | 71.40±24.65 | 0.692 |
| POD1 Prothrombin time (s) | 14.70(12.95-16.08) | 14.10(13.25-14.90) | 0.476 |
| POD3 WBC (× 10^9^/L) (mean, SD) | 10.10±3.09 | 9.96±2.94 | 0.780 |
| POD3 Neutrophil (× 10^9^/L) (mean, SD) | 7.80±2.75 | 9.37±13.46 | 0.333 |
| POD3 Hemoglobin (g/L) (mean, SD) | 122.97±16.51 | 119.70±18.60 | 0.268 |
| POD3 Platelet (× 10^9^/L) (mean, SD) | 169.81±91.71 | 160.88±76.27 | 0.528 |
| POD3 Total Bilirubin (µmol/L) (n, %) | 20.30(15.10-27.68) | 20.50(14.70-28.43) | 0.958 |
| POD3 ALT (U/L) (n, %) | 110.05(78.93-156.25) | 125.85(83.60-166.20) | 0.211 |
| POD3 Albumin (g/L) (mean, SD) | 39.82±18.83 | 36.46±4.19 | 0.148 |
| POD3 Creatinine (μmoI/L) (mean, SD) | 63.04±16.75 | 62.01±20.53 | 0.746 |
| POD3Prothrombin time (s) | 14.65(13.25-16.13) | 14.60(13.20-15.63) | 0.898 |
| POD7 AFP (ng/ml) (n, %) | 2256(138.37-12082.25) | 88.11(6.69-1289.05) | **0.034** |
| POD7 WBC (× 10^9^/L) (mean, SD) | 7.63±2.48 | 8.94±2.55 | **0.002** |
| POD7 Neutrophil (× 10^9^/L) (mean, SD) | 4.77±1.93 | 6.43±4.29 | **0.004** |
| POD7 Hemoglobin (g/L) (mean, SD) | 120.43±16.68 | 121.99±17.49 | 0.588 |
| POD7 Platelet (× 10^9^/L) (mean, SD) | 221.38±101.92 | 202.95±98.04 | 0.273 |
| POD7 Total Bilirubin (µmol/L) (n, %) | 14.60(10.10-21.70) | 17.75(13.43-23.10) | **0.015** |
| POD7 ALT (U/L) (n, %) | 65.60(48.20-87) | 66.30(49.50-93.40) | 0.686 |
| POD7 Albumin (g/L) (mean, SD) | 40.41±7.27 | 38.70±5.27 | 0.106 |
| POD7 Creatinine (μmoI/L) (mean, SD) | 64.56±20.42 | 66.69±15.53 | 0.486 |
| POD7 Prothrombin time (s) | NA | NA | NA |
| POD30 AFP (ng/ml) (n, %) | 46.56(4.87-1430.45) | 8.74(3.33-152.93) | **0.018** |
| POD30 WBC (× 10^9^/L) (mean, SD) | 5.54±2.16 | 6.16±1.73 | 0.123 |
| POD30 Neutrophil (× 10^9^/L) (mean, SD) | 3.17±1.89 | 3.44±1.60 | 0.457 |
| POD30 Hemoglobin (g/L) (mean, SD) | 130.69±16.35 | 132.77±14.43 | 0.519 |
| POD30 Platelet (× 10^9^/L) (mean, SD) | 155.49±79.34 | 186.36±72.55 | 0.055 |
| POD30 Total Bilirubin (µmol/L) (n, %) | 11.70(9.50-16.84) | 10.70(8.90-14.20) | 0.108 |
| POD30 ALT (U/L) (n, %) | 35.10(25.15-48.65) | 29.60(22.45-46.70) | 0.163 |
| POD30 Albumin (g/L) (mean, SD) | 42.36±5.45 | 44.48±7.06 | 0.080 |
| POD30 Creatinine (μmoI/L) (mean, SD) | 67.80±32.47 | 69.18±18.01 | 0.791 |
| POD30 Prothrombin time (s) | 13.75(12.10-14.48) | 12.30(11.80-12.90) | **0.009** |

Abbreviations: LLL: left lateral lobectomy; LH: left hepatectomy; POD: postoperative day; WBC: white blood cell; ALT: alanine aminotransferase; AFP: alpha-fetoprotein.
